# Germ band retraction as a landmark in glucose metabolism during *Aedes aegypti *embryogenesis

**DOI:** 10.1186/1471-213X-10-25

**Published:** 2010-02-25

**Authors:** Wagner Vital, Gustavo Lazzaro Rezende, Leonardo Abreu, Jorge Moraes, Francisco JA Lemos, Itabajara da Silva Vaz, Carlos Logullo

**Affiliations:** 1 Laboratório de Química e Função de Proteínas e Peptídeos and Laboratório de Biotecnologia-CBB-UENF, Av Alberto Lamego, 2000, Horto, CEP 28015-620 Campos dos Goytacazes, RJ, Brazil; 2 Centro de Biotecnologia do Estado do Rio Grande do Sul and Faculdade de Veterinária, UFRGS, Av Bento Gonçalves 9500, CP 15005, 91501-970, Porto Alegre, RS, Brazil; 3 Laboratório de Fisiologia e Controle de Artrópodes Vetores, IOC - Fiocruz, Av. Brasil 4365, CEP 21045-900 Rio de Janeiro, RJ, Brazil; 4 Laboratório de Entomologia, IBEx, Rio de Janeiro, RJ, Brazil; 5 Instituto de Bioquímica Médica - UFRJ/Macaé and Núcleo em Ecologia e Desenvolvimento Sócio-Ambiental de Macaé-NUPEM-UFRJ, Av São José do Barreto s/n, CEP 27971-550, São José do Barreto, Macaé, RJ, Brazil

## Abstract

**Background:**

The mosquito *A. aegypti *is vector of dengue and other viruses. New methods of vector control are needed and can be achieved by a better understanding of the life cycle of this insect. Embryogenesis is a part of *A. aegypty *life cycle that is poorly understood. In insects in general and in mosquitoes in particular energetic metabolism is well studied during oogenesis, when the oocyte exhibits fast growth, accumulating carbohydrates, lipids and proteins that will meet the regulatory and metabolic needs of the developing embryo. On the other hand, events related with energetic metabolism during *A. aegypti *embryogenesis are unknown.

**Results:**

Glucose metabolism was investigated throughout *Aedes aegypti *(Diptera) embryonic development. Both cellular blastoderm formation (CBf, 5 h after egg laying - HAE) and germ band retraction (GBr, 24 HAE) may be considered landmarks regarding glucose 6-phosphate (G6P) destination. We observed high levels of glucose 6-phosphate dehydrogenase (G6PDH) activity at the very beginning of embryogenesis, which nevertheless decreased up to 5 HAE. This activity is correlated with the need for nucleotide precursors generated by the pentose phosphate pathway (PPP), of which G6PDH is the key enzyme. We suggest the synchronism of egg metabolism with carbohydrate distribution based on the decreasing levels of phosphoenolpyruvate carboxykinase (PEPCK) activity and on the elevation observed in protein content up to 24 HAE. Concomitantly, increasing levels of hexokinase (HK) and pyruvate kinase (PK) activity were observed, and PEPCK reached a peak around 48 HAE. Glycogen synthase kinase (GSK3) activity was also monitored and shown to be inversely correlated with glycogen distribution during embryogenesis.

**Conclusions:**

The results herein support the hypothesis that glucose metabolic fate changes according to developmental embryonic stages. Germ band retraction is a moment that was characterized as a landmark in glucose metabolism during *Aedes aegypti *embryogenesis. Furthermore, the results also suggest a role for GSK3 in glycogen balance/distribution during morphological modifications.

## Background

The mosquito *Aedes aegypti *is vector of urban yellow fever and also the main dengue vector [1]. One of the major problems involving dengue transmission is that *A. aegypti *embryos enter dormancy at the end of embryogenesis, surviving and remaining viable for several months inside the egg [2-4]. This extended viability is possible due to the acquisition of embryonic desiccation resistance, a biological mechanism that is believed to involve the formation and maturation of serosal cuticle, a layer covering the embryo [5]. Despite its importance as a vector, little attention is given to *A. aegypti *embryonic development. Taking into account the fact that mosquito populations are becoming resistant to the insecticides currently available for vector control [6], it is imperative to establish new vector control methods. These methods can be developed from a better comprehension of the biology of these insects, since some parts of their life cycle, such as embryogenesis, are still poorly understood.

As a rule, oviparous animals face embryogenesis in the absence of exogenous nutrient supply. In this case, maternal nutrients are packaged into the female gamete (oocytes) during oogenesis [7,8]. In insect oogenesis the oocytes exhibit fast growth, accumulating carbohydrates, lipids and proteins that will meet the regulatory and metabolic needs of the developing embryo [9]. In mosquitoes, the majority of yolk components are synthesized at extraovarian sites, primarily in the female fat body [10-14]. Subsequently, these yolk components are transported via haemolymph and incorporated into the oocytes [15]. The sequential deposition of yolk components was evaluated during oogenesis in *A. aegypti*. Synchronous protein and lipid incorporation into the oocytes occurs in the first 36 h, while rapid glycogen incorporation happens between 36 and 48 h of oogenesis [14].

The current literature provides ample information regarding metabolic events during larval and adult phases of *A. aegypti *[14,16-19]. Nevertheless, in *A. aegypti *embryogenesis, aspects concerning energy metabolism such as the activity of central metabolic pathways (e.g. glycolysis and gluconeogenesis) or the determination of energy reserves to be used have been neglected. In the fruit fly *Drosophila melanogaster *an increase in glycogen content strongly correlated with protein levels in follicles and young embryos has been described [20-22]. Histochemical studies reveal that glycogen is the predominant form of carbohydrate storage in *D. melanogaster *eggs [23]. Additionally, the amount of carbohydrates was shown to decrease from late oocyte stages until after 2 h of embryogenesis, and increases up to the blastoderm stage, during later development [23]. Furthermore, changes in protein content occur in an opposite direction to that determined for the carbohydrate content [22]. Moreover, in *D. melanogaster *glycogen is abundantly stored in the midgut compartment during late stages of embryogenesis [23,24].

Insulin is a key regulator of energetic metabolism in many organisms. It increases glucose transport, glycogen synthesis, diminishes gluconeogenesis, inhibits glycogenolysis, and regulates the expression of various genes [25]. Components of insulin signaling pathway have been discovered to be extremely conserved in organisms as distantly related as humans, *D. melanogaster *and *Caenorhabditis elegans *[26]. In *A. aegypti*, upstream components of insulin signaling pathway, as phosphatidylinositol 3-kinase (PI3K) [27] and protein kinase B (AKT) [28], have been identified and correlated with glucose metabolism. Glycogen synthase kinase-3 (GSK3), is a serine-threonine kinase present as two highly homologous forms, GSK3a and GSK3b, was first identified based on its action towards glycogen synthase (an enzyme involved in glycogen biosynthesis), and is also considered a downstream component of insulin signaling cascade [29,30]. GSK3 is now recognized as a key component of a surprisingly large number of cellular processes. A previous study on the cattle tick *Rhipicephalus microplus *conducted by our research group revealed that GSK3 activity was correlated with diminished glycogen content in eggs during embryogenesis [31].

In this paper we correlated different biochemical parameters of glucose metabolism with morphological changes that take place during *A. aegypti *embryo development. It was also demonstrated that glucose and glycogen levels are closely correlated with activity and transcription levels of GSK3 during embryogenesis. It suggests a highly conserved participation of GSK3 in glycogen metabolism in arthropod embryogenesis. To the best of our knowledge there is no work describing either function or activity of GSK3 in mosquitoes, or GSK3 involvement in glycogen metabolism.

## Results

### Embryonic development

**Figure 1:**
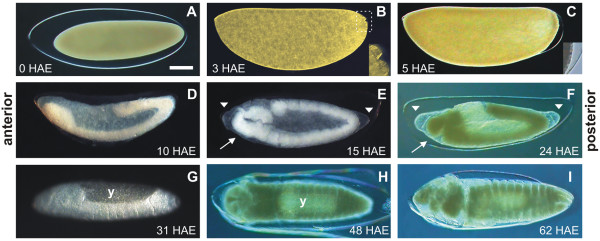
***Aedes aegypti *embryogenesis at 28°C**. (**A**) 0 h after egg laying (HAE) embryo, detached from the endochorion. (**B**) 3-HAE embryo at the syncytial blastoderm stage. Insert shows the pole cells outside the blastoderm. (**C**) 5-HAE embryo, at the cellular blastoderm stage. Insert shows ventral-posterior region of the blastoderm detached from the endochorion and cell boundaries. (**D**) 10-HAE embryo in the middle of germ band extension. (**E**) 15-HAE embryo at the beginning of germ band retraction. (**F**) 24-HAE embryo at the germ band retraction stage. (**G**) 31-HAE embryo, at dorsal closure stage. Focal plane is located inside the embryo at the embryo-yolk junction region. (**H**) 48-HAE embryo at late organogenesis stage. Larvae segmentation is partially evident. (**I**) 62-HAE embryo at the end of embryogenesis showing the head, three fused thoracic segments, eight abdominal segments and the respiratory siphon and associated structures. In **B-G**, dorsal side is up. Scale bar = 100 μm. Arrow: cephalic region segmented. Arrowhead: serosal cuticle limits, detached from the endochorion. y: yolk. **A-C, F, H, I**: DIC microscopy. **C insert**: Bright field. **D, E, G**: Stereoscope.

In order to determine the timing of major morphological landmarks during *A. aegypti *embryogenesis at 28°C, embryos in distinct stages were clarified [32] and observed (Figure 1). Zero hours after egg laying (HAE), eggs have just been fertilized and the embryos are detached from the surrounding endochorion (Figure 1A). Three HAE embryos are at the syncytial blastoderm stage, with the pole cells positioned outside the blastoderm (Figure 1B) [2,33], while 5 HAE embryos are right before or at the cellular blastoderm stage (Figure 1C) [33]. Ten and 15 HAE embryos are in the middle of germ band extension and at the beginning of germ band retraction, respectively (Figure 1D, 1E) [5]. Twenty-four HAE embryos are in the middle of germ band retraction (Figure 1F) [4]. Thirty-one HAE, embryos are at the dorsal closure stage (Figure 1G), while 48 HAE embryos are at late organogenesis stage [34], with evident larvae segmentation (Figure 1H). The embryonic development at 28°C is completed 61.6 HAE [35]. Accordingly, 62 HAE embryos show all the features of larvae ready to hatch (Figure 1I) [4,35].

### Glycolytic pathway increases after germ band formation in *A. aegypti *embryos

**Figure 2:**
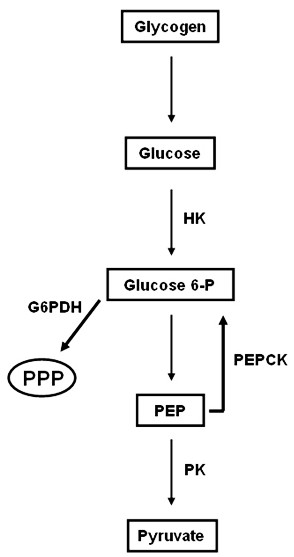
**Representative scheme for pathways of glucose metabolism**. The scheme is based on enzyme activities and metabolites quantification evaluated during *Aedes aegypti *embryogenesis. HK - Hexokinase, G6PDH - Glucose 6-phosphate Dehydrogenase, PK - Pyruvate Kinase, PEPCK - Phosphoenolpyruvate Carboxykinase, PEP - Phosphoenolpyruvate and PPP - Penthose-Phosphate Pathway.

**Figure 3:**
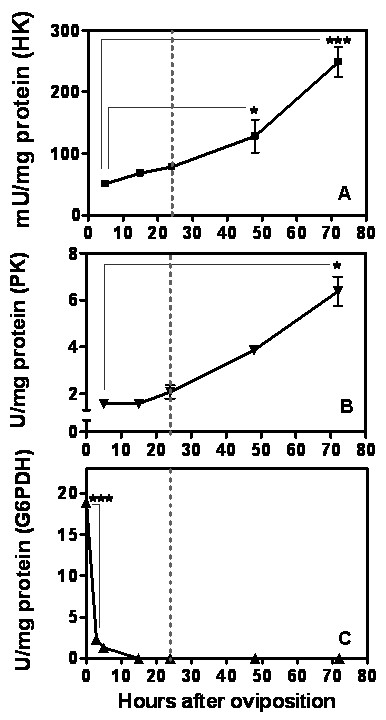
**HK, PK and G6PDH activities during *A. aegypti *embryogenesis**. The HK (A), PK (B) and G6PDH (C) activities were measured in egg homogenates on different hours of embryo development. Each experiment was replicated three times and error bars represent standard deviation of sample means. (*p < 0.05; ***p < 0.001, ANOVA). The dashed line indicates the germ band retraction stage 24 HAE.

The glycolytic pathway transforms glucose into pyruvate obtaining ATP during this process (Figure 2). In *A. aegypti *embryogenesis the glycolytic pathway was evaluated determining the enzymatic activity of hexokinase (HK) and pyruvate kinase (PK) (Figure 3). HK and PK are respectively the initial and final steps of glycolysis and both are regulatory enzymes that control the flux of glycolysis. The PK and HK patterns of activity are positively correlated throughout embryogenesis. This demonstrates that the activity of these enzymes occurs concomitantly, exerting a connected action at this phase of mosquito embryo development. HK and PK activities are low during the first 15 h of embryogenesis (Figure 3A and 3B).

### Glucose 6-phosphate is mainly destined to pentose-phosphate pathway before the germ band formation in *A. aegypti *embryos

The pentose-phosphate pathway, which produces NADPH and ribose 5-phosphate (Figure 2), was investigated during embryogenesis by determining the activity profile of glucose 6-phosphate dehydrogenase (G6PDH), the rate limiting step of this pathway [36]. The very beginning of mosquito embryogenesis is marked by high levels of G6PDH activity 0 HAE, followed by a drastic decrease in the first 5 h of embryo development (Figure 3C). G6PDH activity was no longer detected 15 HAE (beginning of germ band retraction).

### *A. aegypti *embryo GSK3 sequence analysis

RT-PCR from cDNA obtained from *A. aegypti *embryos between 0 and 9 HAE (see Item 2.12) using degenerated primers for GSK3 generated an approximately 600-bp product (data not shown). The cloned fragment was sequenced and showed a 100% identity with an *A. aegypti *GSK3 sequence deposited in GenBank (ascension number: DQ440045.1) and *A. aegypti *Gene Index (TC35709). Then, the full-length mosquito GSK3 sequence was compiled from the data obtained by sequencing the cloned fragment and database sequences analysis. The deduced amino acid sequence of the *A. aegypti *GSK3 was compared with orthologs from a number of other species (Additional File 1). The partial AeGSK3 cDNA is 3,690 bp long and had an open reading frame of 1,476 bp, beginning with the first ATG codon at position 97 bp and with the stop codon at position 1,572 bp.

The AeGSK3 sequence was analyzed with BLAST and ScanProsite tools. The Protein kinase ATP-binding region (residues Ile-60 - Lys-184) and Serine/Threonine protein kinase active-site signatures (residues Ile-175 - Leu-187), which are important for determining its biological proprieties, were found (Additional File 1).

### The control of glycogen metabolism in *A. aegypti *embryos

**Figure 4:**
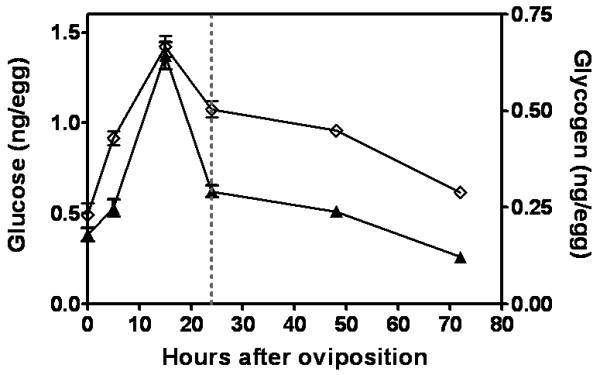
**Glucose and glycogen levels during *A. aegypti *embryogenesis**. The glucose (open lozenge) and glycogen (black up-pointing triangle) concentration were measured in egg homogenates on different hours of embryo development. Each experiment was replicated six times and error bars represent standard deviation of sample means. The dashed line indicates the germ band retraction stage 24 HAE.

**Figure 5:**
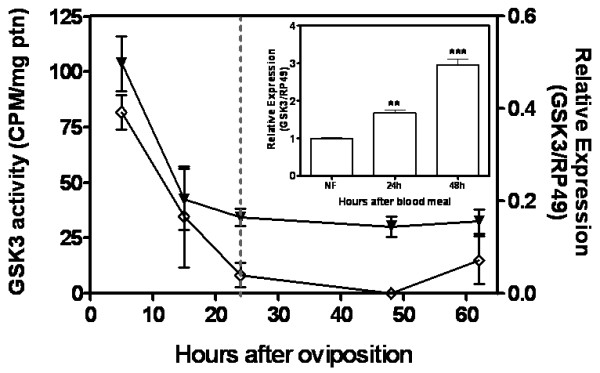
**Glycogen Synthase Kinase-3 (GSK3) activity and relative expression during *A. aegypti *embryo development**. The GSK3 activity (open lozenge) and GSK3 transcripts levels (black down-pointing triangle) (normalized by *rp49 *cDNA) were measured on different hours of embryo development. Each experiment was replicated three times and error bars represent standard deviation of sample means. Insert shows GSK3 transcripts levels in ovaries of *A. aegypti *females dissected in different hours after blood meal (**p < 0.01; ***p < 0.001, ANOVA). The dashed line indicates the germ band retraction stage 24 HAE.

Glycogen metabolism in *A. aegypti *embryogenesis was investigated by determining glycogen and glucose content (Figure 4), and glycogen synthase kinase (GSK3) activity and transcription (Figure 5). Total glycogen and glucose amount in eggs increased between 0 and 15 HAE. After that, glycogen and glucose content greatly decreased until the end of embryogenesis (Figure 4). The level of GSK3 activity decreases from 5 HAE to 24 HAE (Figure 5), being inversely correlated with glycogen distribution from 5 to 15 HAE. From 24 HAE until the end of embryogenesis GSK3 activity remains at low levels. To identify the profile of GSK3 mRNA transcription, cDNAs obtained from eggs collected at different embryogenesis stages and from blood-fed and not blood-fed females ovaries were analyzed by qPCR. Figure 4 shows the relative amount of *GSK3 *mRNA, normalized by *rp49 *mRNA (a constitutively expressed house-keeping gene). Embryos at different stages of development were evaluated over the course of embryogenesis between 0 HAE (designated as calibrator) (data not shown) and 62 HAE. The GSK3 gene expression decreased sharply between 0 and 24 HAE, from 100% to 16% of initial relative amount of GSK3 mRNA (Figure 5). After this moment (middle of germ band retraction) the relative amount of GSK3 mRNA remained stable, around 15% of its initial value, until the end of embryogenesis.

GSK3 mRNA transcripts are present in ovaries from blood-fed and not blood-fed females (Figure 5-insert). In contrast to embryos, GSK3 relative expression increased significantly (nearly 1.5 times), when comparing ovaries of not blood-fed females (designated as calibrator) and ovaries of females 24 h after blood meal. In ovaries of females 48 h after blood meal, GSK3 relative expression increased significantly by 3 times, when compared to not blood-fed females.

### Gluconeogenesis increases after germ band formation in *A. aegypti *embryos

**Figure 6:**
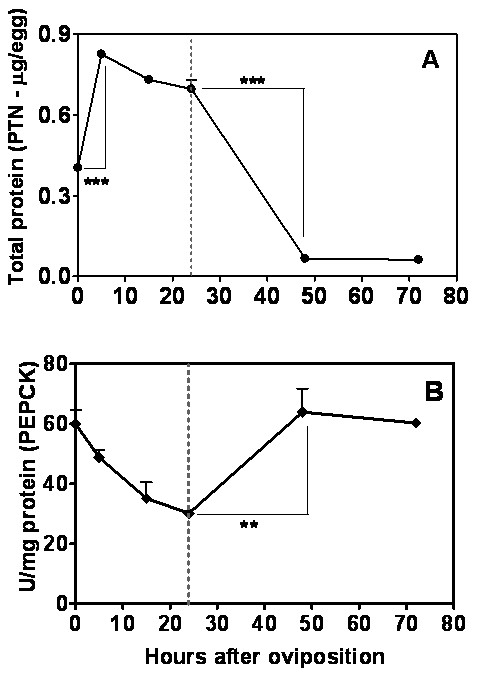
**Gluconeogenesis pathway during *A. aegypti *embryogenesis**. (A) The protein concentration was measured in egg homogenates. (B) The PEPCK activity was measured in egg homogenates. Each experiment was replicated three times and error bars represent standard deviation of sample means. (**p < 0.01; ***p < 0.001, ANOVA). The dashed line indicates the germ band retraction stage 24 HAE.

The gluconeogenesis consists of the formation of glucose from noncarbohydrate precursors, such as the products of lipids and proteins breakdown (Figure 2). The evaluation of gluconeogenesis during embryogenesis was carried out by correlating protein and glucose content in eggs (Figures 6A and 4, respectively), and the activity of phosphoenolpyruvate carboxykinase (PEPCK) (Figure 6B), the key enzyme of this pathway. Glucose content increased until 15 HAE, and then was gradually reduced towards embryogenesis completion (Figure 4). Total protein content in eggs increased until 5 HAE, at the stage of cellular blastoderm formation. From then until the middle of germ band retraction (24 HAE), protein content was maintained and then declined drastically between 24 and 48 HAE, and remained at low levels until the end of embryogenesis (Figure 6A). PEPCK activity decreased continuously until 15 HAE, being inversely correlated with glucose content up to that point (compare Figures 4 and 6B). PEPCK activity subsequently doubled between 24 and 48 HAE, remaining at high levels until the end of embryogenesis, concomitantly with a significant decrease in protein content (Figure 6).

## Discussion

In a previous work with the cattle tick *R. microplus *embryos our group demonstrated a correlation between the kinetics of egg energetic components mobilization and the morphological changes occurring during early embryogenesis [37]. In this work, stages of *A. aegypti *embryogenesis were visualized in clarified embryos (Figure 1) and compared with the metabolic modifications related to glucose metabolism throughout embryogenesis. Farnesi et al. [35] demonstrated that *A. aegypti *embryonic development is completed around 62 HAE when the specimens are reared at 28°C. According to our analysis, *A. aegypti *exhibits the same pattern of embryo formation as described for *D. melanogaster *[38] and for another mosquito, *Anopheles albitarsis *[39].

Acquisition of drug resistance by insects vectors of diseases is a major global health problem, due to a rapid selection of insects resistant to compounds used as conventional insecticides [6]. In fact, insecticide resistance is the fastest case of evolutive adaptation ever observed and it is extensively documented [40]. The discovery of new drugs aimed at working as insecticides demand a high investment in research and these drugs might have a short lifetime efficacy due to the acquisition of resistance by insects. Therefore, the discovery of new drugs that can be used as new insecticides must be a constant process. A strategy for development of new control methods can rely on the characterization of the existing metabolic pathways in insects, in order to identify targets present in the insect but not it the host. This approach was successfully employed regarding chitin metabolism and the moulting process, with juvenile hormone analogues [41]. So, further studies are needed to confirm the viability of this strategy to new metabolic pathways and distinct life stages, such as the embryo.

Hexokinase and Pyruvate Kinase catalytic activities were detected throughout embryogenesis, and presented increase from 24 HAE on which suggest that glycolysis is intensified after germ band retraction stage (Figure 3) remaining elevated until the end of embryogenesis. At the very beginning of embryogenesis, however, the glucose 6-phosphate (G6P) produced by hexokinase would, most likely, be driven to the pentose-phosphate pathway (PPP), due to the high activity of G6PDH, 0 HAE. After, G6PDH catalytic activity declined abruptly between 0 and 5 HAE, and was nearly null 15 HAE. This result suggests an intense participation of PPP in the initial part of *A. aegypti *embryogenesis. Our observations support an abrupt shift in glucose fate at the moment of cellular blastoderm formation (CBf), which occurs 5 HAE. Before CBf, *A. aegypti *embryos must sustain a high synthesis of nucleic acids due to the intense nuclei division for the formation of the syncytial blastoderm occurring prior to CBf [33]. At this moment the PPP would supply ribose 5-phosphate units for the synthesis of nucleotides. The NADPH produced by PPP would also be used for phospholipids necessary for blastoderm cellularization happening 5 HAE. A similar increase in G6PDH activity prior to CBf was observed during embryonic development of the hard tick *R. microplus *[42].

Total protein content in *A. aegypti *eggs was significantly increased between 0 and 5 HAE. Elevations in protein levels were previously observed in *D. melanogaster *embryos, and an early elevation in protein content during embryogenesis can be explained by maternal mRNA driven protein synthesis (transcription of maternal message) [22]. Our data suggest that such protein biosynthesis in *A. aegypti *embryos could be supported by concomitant generation of reducing potential (NADPH) by the highly active PPP.

An increase in glucose and glycogen content was observed during the first hours of embryogenesis (between 0 and 15 HAE). The peak of glucose, observed 15 HAE, might be important for the synthesis of the chitinized serosal cuticle that happens at this very moment [5]. This hypothesis is supported by evidences which indicate that the amino sugar pathway (leading to chitin formation) is upregulated at this development stage (unpublished data). Therefore, chitin production (employed on the construction of the serosal cuticle), might be a sink for glucose consumption at this stage of development. Further experiments will confirm this hypothesis. Additionally, chitin production used for organogenesis and synthesis of the larval cuticle at late embryogenesis, starting from 31 HAE [5,34], might also be consuming glucose, although at this moment no peak of glucose is observed. Previous works in *Drosophila *embryogenesis also identified an increase in glycogen and carbohydrates content, which nevertheless occurred at the earlier developmental stage of blastoderm formation [21,22]. It has been previously observed that glycogen accumulates in embryos of the cattle tick *R. microplus *in parallel with an increase in PEPCK catalytic activity. It was suggested that glycogen biosynthesis could be supported by concomitant gluconeogenesis [42]. On the other hand, during *A. aegypti *embryogenesis, PEPCK activity declined between 0 and 24 HAE (Figure 6B), suggesting the availability of other carbohydrates source in insects, such as trehalose [43-46], which should be mobilized and converted into glucose and/or glycogen.

After the progressive drop until 24 HAE, a significant increase in PEPCK catalytic activity was observed between 24 and 48 HAE, concomitant with a consistent reduction in total protein content, the main gluconeogenic substrate (Figure 6A and 6B). Taken together, these results suggest a correlation between protein content, gluconeogenic pathway, and morphogenetic modifications that occur during germ band retraction (24 HAE), dorsal closure stage (31 HAE) and late organogenesis (48 HAE) (compare Figures 6 and 1). In this scenario, glycogen would be produced using proteins as substrate, during the whole organogenesis process that takes place after germ band retraction [34,4]. Yamazaki and Yanagawa [23] also reported an oscillation in glycogen distribution during *D. melanogaster *embryogenesis. Conversely, after being almost totally consumed, glycogen content increased at the late stages of embryo formation (when organogenesis takes place) and accumulates in embryo midgut.

Our previous work demonstrated that glycogen distribution throughout *R. microplus *embryogenesis was inversely associated with GSK3 activity [31]. Due to its role on glycogen synthesis regulation, both GSK3 activity and its relative expression were determined during *A. aegypti *embryogenesis (Figure 5). We observed that GSK3 activity was directly related to GSK3 transcript levels in *A. aegypti *embryos. Both GSK3 activity and gene transcription dropped between 5 and 24 HAE. Furthermore, GSK3 activity was inversely related to the glycogen content in the interval 5 to 15 HAE (Compare with Figures 4 and 5) suggesting that glycogen accumulation in eggs can be regulated by GSK3 activity during cellular blastoderm formation and germ band extension (Figure 1). GSK3 activity is classically described as negatively regulated by insulin signaling pathway [28]. We suggest that such regulation may be conserved in invertebrate organisms, like mosquitoes. In this perspective, a high insulin signal would be present during the first 15 h, leading to drop in GSK3 activity and increasing glycogen formation (GSK3 activity inhibits glycogen synthase, the enzyme responsible for glycogen formation). In fact, three out of the eight genes encoding insulin-like peptides in *A. aegypti *were previously reported to be expressed in eggs [47]. On the other hand, it has been already demonstrated that GSK3 is involved with embryo dorsoventral axis formation in *D. melanogaster *[48-50] and *Xenopus *[51-53], rather as a component of *Wnt *signaling pathway than as regulator of the metabolism of glycogen. Further studies will be necessary to describe whether GSK3 plays a role on the control of cell differentiation and embryo polarity patterning during mosquito embryogenesis. Additionally, we compared the AeGSK3 cDNA amino acid sequence (ascension number: DQ440045.1) and it was revealed a high homology indexes for vertebrates and invertebrates species (Additional File 1).

Several authors have reported the effect of mosquito/insect blood meal on gene expression [54-56]. GSK3 was upregulated in ovaries from bloodfed *A. aegypti *females, when compared to unfed females. Hence, one must consider a possible role for GSK3 during mosquito oogenesis. In *A. aegypti *mosquito, an insulin-like peptide was shown to regulate oocyte maturation and metabolism [19]. Monosaccharides were previously measured in mature oocytes well after follicular growth, and glycogen appeared during or even after oocyte chorionation [14]. It has been suggested that chorionated oocytes must retain enzymes able to synthesize glycogen long after the end of oogenesis, presumably due to activation of glycogen synthase enzyme (GS). Additionally, Briegel *et al*. [14] postulate that late accumulation of glycogen in *A. aegypti *developing oocytes could be due to GS activity inhibition. The increase in GSK3 transcript levels observed during *A. aegypti *oogenesis suggests its activation as a mechanism to inhibit GS activity and regulate glycogen incorporation kinetics in developing oocytes. Therefore, from late oogenesis up to the initial developmental stages GSK3 activity and expression are high and glycogen content is relatively low. From 24 HAE on, glycogen metabolism appears to switch in order to reduce its biosynthesis and accumulation (Figure 4). Coincidentally, this same period of development is marked by a strong reduction in both GSK3 activity and expression (Figure 5).

## Conclusions

**Figure 7:**
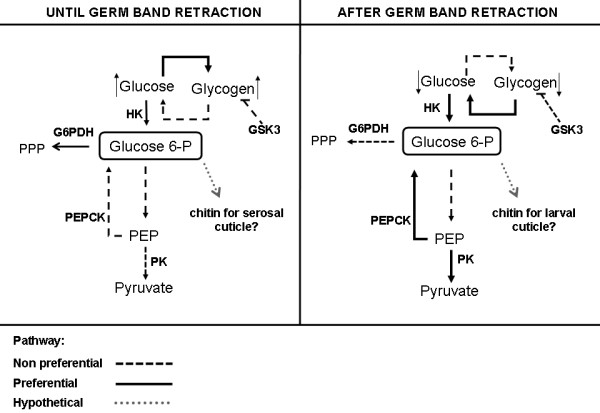
**Proposed scheme for glucose metabolism during the *A. aegypti *embryo development**. The components represented herein were determined as described in Materials and Methods. HK: Hexokinase, G6PDH: Glucose 6-phosphate Dehydrogenase, PEP: Phosphoenolpyruvate, PEPCK: Phosphoenolpyruvate Carboxykinase, PK: Pyruvate Kinase, PPP: Pentose-Phosphate Pathway and GSK3: Glycogen Synthase Kinase 3.

The results presented here demonstrated that glucose metabolism is closely correlated to *A. aegypti *developmental embryonic stages. Furthermore, germ band retraction is a landmark regarding both glucose and glycogen metabolism. It is important to stress that the shift in glucose metabolism is related with the cellular processes that are taking place before and after germ band retraction. We intend to study these processes in detail in the future. The results observed in the present study are schematically represented together in Figure 7. Nevertheless, further elucidation of these phenomena would lead to a better understanding of the regulatory mechanisms in glucose metabolism during *A. aegypti *embryogenesis.

## Methods

### Mosquito maintenance

*Aedes aegypti *(Rockefeller strain) were reared constantly in the laboratory. Larvae were fed with rat food, and adults were fed *ad libitum *with 10% (w/v) sucrose. For egg production, female mosquitoes were blood-fed on a mouse.

To perform oogenesis assays, females were kept at 28°C in a BOD humid chamber with a 12·h:12·h, light:dark cycle for a period of 24 and 48 h after blood meal.

### Synchronous Egg-laying

This method was performed as described previously [5], but oviposition lasted 30 minutes. Hours after egg laying (HAE) were considered as the age assigned to a sample starting from after the 30-min egg laying period. Eggs were kept humid/wet at 28°C until the end of embryogenesis or collected at indicated HAE. For all biochemical assays embryo development was interrupted by freezing the samples in liquid nitrogen.

### Egg homogenates

Egg homogenates were prepared by grinding eggs in appropriate extraction buffer (80 mg of eggs/mL of buffer). Then, homogenates were centrifuged at 200 × g for 10 min to sediment insoluble eggshell fragments. Supernatant aliquots were assayed. The composition of each extraction buffer is described on items 2.4 - 2.11. In order to determine the content of metabolites (glucose, glycogen and total protein) the average weight of eggs of each age used was estimated (data not shown).

Egg homogenates for determination of total protein and enzymatic activities were prepared in the presence of the following protease inhibitors: 1 μM phenylmethylsulfonyl fluoride (PMSF), 1 mM ethylenediamine tetraacetic acid (EDTA), 10 μM iodacetamide, 1 μM pepstatin A and 10 μM leupeptine.

### Determination of glucose content

Eggs were homogenized in 200 mM phosphate buffered saline (PBS) pH 7.4. Glucose content was enzymatically quantified by glucose oxidase (glucox^® ^enzymatic Kit for glucose dosage; Doles, inc.). After 30 min incubation at 37°C the samples were read at 510 nm in a Shimadzu U1240 spectrophotometer, according to the manufacturer's instructions.

### Determination of glycogen content

Egg glycogen content was determined as described elsewhere [42]. Egg homogenates were prepared in 200 mM sodium acetate, pH 4.8, and supernatant aliquots (five replicates from each sample) were incubated with 1 unit of α-amyloglucosidase (Sigma Chemicals) for 4 h at 40°C. The newly generated glucose was enzymatically determined by glucose oxidase as described for glucose content determination. Free glucose was subtracted from samples without α-amyloglucosidase. Glycogen content was determined using a standard curve submitted to the same conditions.

### Determination of Protein Content

Eggs were homogenized 0, 3, 5, 15, 24, 48, 62 and 72 HAE in 200 mM phosphate buffered saline (PBS) pH 7.4. The protein content of samples was determined according to the Lowry method [57], using bovine serum albumin as standard.

### Hexokinase (HK) activity Assay

Eggs were homogenized in extraction buffer containing 20 mM Tris-HCl, pH 7.5 with 6 mM MgCl_2 _5, 15, 24, 48 and 72 HAE. Supernatant aliquots (in triplicate) were assayed in 20 mM Tris-HCl pH 7.5 containing 6 mM MgCl_2_, 1 mM ATP, 0.5 mM NAD^+ ^and 2 mM glucose. HK catalytic activity was measured by adding *Leuconostoc mesenteroides *glucose 6-phosphate dehydrogenase (Sigma-Aldrich Chemicals) (Worthington Code: ZF or ZFL) dissolved at a concentration of 1 UI/mL in the above Tris-MgCl_2 _buffer [58]. The production of β-NADH was monitored at 340 nm in a Shimadzu U1240 spectrophotometer using a molar extinction coefficient of 6.22 M^-1^, as described by Worthington [59].

### Pyruvate kinase (PK) activity Assay

Eggs were homogenized in extraction buffer containing 20 mM Tris-HCl pH 7.5 with 5,5 mM MgCl_2 _5, 15, 24, 48 and 72 HAE. Supernatant aliquots (in triplicate) were assayed in 1 mL of 20 mM Tris-HCl pH 7.5 containing 5.5 mM MgCl_2_, 1 mM ADP, 0.4 mM NADH, 1 unit/mL lactate dehydrogenase and 1 mM phosphoenolpyruvate. The β-NADH consumption was monitored at 340 nm in a Shimadzu U1240 spectrophotometer using a molar extinction coefficient of 6.22 M^-1 ^as described by Worthington [59].

### Glucose-6-phosphate dehydrogenase (G6PDH) activity Assay

Egg homogenates were homogenized in extraction buffer containing 55 mM Tris-HCl pH 7.8 0, 3, 5, 15, 24, 48 and 72 HAE. Supernatant aliquots (in triplicate) were assayed in 1 mL of 55 mM Tris-HCl, pH 7.8 containing 6 mM MgCl_2_, 100 mM glucose 6-phosphate and 0.5 mM β-NADP^+^. The reaction was started with sample addition. The formation of β-NADPH was monitored at 340 nm in a Shimadzu U1240 spectrophotometer during 5 min, using a molar extinction coefficient of 6.22 M^-1 ^as described by Worthington [59].

### Phosphoenolpyruvate carboxykinase (PEPCK) activity Assay

Egg homogenates were homogenized in extraction buffer containing 100 mM HEPES buffer, pH 7.0 0, 5, 15, 24, 48 and 72 HAE. Supernatant aliquots (in triplicate) were assayed in 400 μL of 100 mM HEPES buffer pH 7.0 containing 10 mM MnSO_4_, 100 mM KHCO_3_, 2 mM reduced glutathione, 10 mM PEP, 0.2 mM NADH, and 24 units of malate dehydrogenase (Sigma Chemicals). The reaction started by the addition of 10 μL 2.5 mM inosine diphosphate (IDP) and the consumption of β-NADH was monitored at 340 nm and PEPCK activity was determined as described by Petersen et al. [60].

### Glycogen Synthase Kinase activity (GSK3) Assay

Egg homogenates were homogenized 5, 15, 24, 48 and 62 HAE in 20 mM Tris-HCl buffer pH 7.4 with 1 mM ammonium molibdate, 1 μg/mL heparin, 1 μM phenylmethylsulfonyl fluoride (PMSF), 1 mM ethylenediamine tetraacetic acid (EDTA), 10 μM iodacetamide, 1 μM pepstatine A and 10 μM leupeptine. Total GSK3 was immunoprecipitated from egg homogenates supernatant aliquots (100 μg of protein) (in triplicate) with anti-GSK3 commercial antibody (Sigma-Aldrich Chemicals). The immuno-complex was captured with protein A-agarose suspension (Sigma-Aldrich Chemicals) by incubating the mixture at room temperature with gentle agitation for 20 min. The resin was collected by centrifugation, washed three times and ressuspended in reaction buffer [20 mM Tris-HCl, pH 7.5, 10 mM MgCl_2_, 5 mM dithiothreitol, 1 mM ammonium molybdate, 1 μg/mL heparin and 50 μM CREB phosphopeptide (Calbiochem). GSK3 activity was determined by incubating the suspension with 100 μM γ-[P32]-ATP (500-3000 CPM/pmol) at 37°C for 30 minutes [61]. After incubation, supernatant aliquots (in quintuplicate) of the supernatant were spotted onto Whatman P81 phosphocellulose paper strips. The strips were washed three times with phosphoric acid solution (75 mM), dried and immersed in scintillation liquid for radioactivity count determination on a 1600TR TRICARB-Packard. The activity was determined as the amount of GSK3 required to catalyze the transfer of 1 pmol of phosphate to CREB Phosphopeptide in 1 min at 30°C.

### RNA isolation and reverse transcription (RT) for GSK3 cloning

Messenger RNA (mRNA) was extracted from embryos collected at 0, 6 and 9 HAE with QuickPrep Micro mRNA Purification kit (Amersham Biosciences), according to the manufacturer's instructions. Approximately 10 ng of mRNA from each sample were reversely transcribed with the First Strand cDNA Synthesis Kit (Amersham Biosciences) using an oligo-dT primer, according to manufacturer instructions.

### PCR and cloning

Primers for GSK3 were designed as degenerated primers based on conserved regions in previously known sea urchin GSK3 cDNA sequence [62]. The pair of degenerated primers used to amplify *A. aegypti *GSK3 is (5'-GTIGCIATHAARAARGTIYTICARGAY-3', and 5'-YTTRWRYTCIRTRTARTTIGGRTTCAT-3') (see Additional File 2). cDNA obtained from embryos (see Item 2.12) was used as templates for PCR. The PCR reactions were performed as follows: 94°C for 5 min, 40 cycles of denaturation at 94°C for 1 min, annealing at 45°C for 1 min, and elongation at 72°C for 1 min, followed by a 10 min extension at 72°C and cooling at 4°C.

PCR products were analyzed by agarose gel electrophoresis, purified with the Wizard SV Gel and PCR clean up systems (Promega) and cloned into pGEM-T Easy vector (Promega). Both strands were sequenced with a BigDye Terminator v3.0, model ABI 377XL sequencer (Applied Biosystems).

### Sequence analysis

Nucleotide sequence identity was performed using the BLAST program (GenBank, NCBI). Amino acid alignment and analysis of GSK3 similarity from selected species was performed using the Clustal W multiple sequences alignment program and BioEdit version 7.0.5.2 software program [63]. The presence of conserved patterns was determined using InterProScan [64].

### Embryo morphology analysis

Eggs obtained from synchronous egg laying were fixed and clarified according to Trpiš (1970) 0, 3, 5, 10, 15, 24, 31, 48 and 62 HAE. This technique fixes the embryo while making the eggshell transparent. Clarified embryos were observed with an Axiophoto microscope (Zeiss) with bright field and DIC and a Stereo Discovery V.12 stereoscope (Zeiss). The embryonic stages were identified according to Raminani and Cupp [33], Clements [4], Rezende *et al*. [5] and Farnesi *et al*. [35]. For every time point at least 150 embryos were clarified and examined. Images were captured from embryos showing representative morphologies of each time point.

### GSK3 relative expression by qPCR

To evaluate GSK3 mRNA expression, total RNA was extracted from the ovary of sucrose-fed females and blood-fed females (24 and 48 h after blood meal) and eggs 0, 5, 15, 24, 48 and 62 HAE. Mosquitoes were washed with 50% ethanol and rinsed with PBS prior to ovary dissection under a microscope. Total RNA was extracted with Trizol reagent (Invitrogen) according to the manufacturer's instructions. RNA quantity and quality were estimated by spectrophotometry at 260/280 nm. Two micrograms of total RNA was reverse-transcribed at 37°C using the High-capacity cDNA Reverse Transcription kit with random primers according to the manufacturer's recommendations (Applied Biosystems). Amplification was performed on LightCycler 1.5 capillary platform (Roche). Serial dilutions of cDNA were used for calibration curve preparation. Reaction efficiencies between 85 and 100% were determined from calibration curves for each set of primers in 10-μL reactions. The primers utilized for specific GSK3 expression were 5'-CGTACATCTGCTCGCGATAC-3'(forward) and 5'-GGATGCGTACTAGCCGAATT-3' (reverse) (Additional File 2). Relative expression was determined by using the Cp values from each run on Relative Expression Software Tool-REST [65], using primers for the constitutive gene *rp49*, used as a reference gene [66]. Values from 0 HAE and ovary from not blood-fed females were designated as calibrators for the expression of relative amount of GSK3 mRNA in embryogenesis and oogenesis, respectively.

## Abbreviations

G6P: Glucose 6-phosphate; PTN: protein; GSK3: Glycogen Synthase Kinase 3; PEPCK: Phosphoenolpyruvate Carboxykinase; HAE: Hours After Egg laying; HK: Hexokinase; PK: Pyruvate Kinase; G6PDH: Glucose 6-phosphate Dehydrogenase; RT: reverse transcriptase; CREB: cAMP-response element-binding protein.

## Authors' contributions

WV and CL designed the experiments and wrote the paper. GLR performed the experiments related to *A. aegypti *embryogenesis morphology. LA and JM performed the experiments related to energetic metabolism. FJAL and ISVJ advised the experiments using mosquitoes and molecular biology. All authors read and approved the final manuscript.

## Supplementary Material

Additional file 1 **GSK3 protein sequence is evolutionary conserved between *A. aegypti *and selected organisms**. Sequence alignment of *Ae*GSK3 and percentage of identical residues between *A. aegypti *and the other respective organisms indicating some conserved regions. Click here for file

Additional file 2 ***A. aegypti *GSK-3 complete cDNA sequence and primer annealing positions**. Representation of the forward and reverse degenerated primers used to clone *Ae*GSK-3 and the primers used for qPCR. Click here for file
