# Supplementary material for: Germ band retraction as a landmark in glucose metabolism during Aedes aegypti embryogenesis
Source: BMC Dev Biol. 2010 Feb 25;10:25. doi: 10.1186/1471-213X-10-25 (PMC2838828; doi:10.1186/1471-213X-10-25)
Supplement: Additional file 1 — GSK3 protein sequence is evolutionary conserved between A. aegypti and selected organisms. Sequence alignment of AeGSK3 and percentage of identical residues between A. aegypti and the other respective organisms indicating some conserved regions. [file 1471-213X-10-25-S1.DOC]

* 20 * 40 *
*A. aegypti* : MSGRPRTTSFAEGNKTQNYP-VMGGMKII-----------SKDGSKVTTV : 38
*M. musculus* : ............SC.PVQQ.SAF.S...SR----------D......... : 40
*H. sapiens* : ............SC.PVQQ.SAF.S...SR----------D......... : 40
*D. rerio* : ............SC.PVPQ.SAF.S...SR----------D......... : 40
*A. gambiae_.*: -----------------------------------------......... : 9
*A. mellifera*: ..S........DCTNAPSN.-P......SSHPAGGVTLKKD...N..... : 49

 60 * 80 * 100
*A. aegypti* : VATAGQGPDRPQEVSYTDTK**VIGNGSFGVVFQATLCDTGELVAIKK**VLQD : 88
*M. musculus* : ...P.............................K................ : 90
*H. sapiens* : ...P.............................K................ : 90
*D. rerio* : ...P.............................K................ : 90
*A. gambiae*  : ...P.............................K................ : 59
*A. mellifera*: ...P.A.........................L.K....E........... : 99

 * 120 * 140 *
*A. aegypti* : KRFKNRELQIMRRLEHCNIVKLKYFFYSSGDKKDEVYLNLVLEYIPETVY : 138
*M. musculus* : ..............D...............E...........D....... : 140
*H. sapiens* : ..............D...............E...........D....... : 140
*D. rerio* : ..............D...........................D....... : 140
*A. gambiae*  : ..............................E................... : 109
*A. mellifera*: .................................................. : 149

 160 * 180 * 200
*A. aegypti* : KVARYYAKNKQTIPINFIRLYMYQLFRSLAYIHSLG*ICHRDIKPQNLLL*D : 188
*M. musculus* : ....H.S.A......I..................F............... : 190
*H. sapiens* : ....H.S.A......I..................F............... : 190
*D. rerio* : ....H.S.A......V..................F............... : 190
*A. gambiae* : ....H.....L....................................... : 159
*A. mellifera*: ....H.N.S......................................... : 199

 * 220 * 240 *
*A. aegypti* : PETAVLKLCDFGSAKQLLHGEPNVSYICSRYYRAPELIFGAINYTTKIDV : 238
*M. musculus* : .D................R......................T....S... : 240
*H. sapiens* : .D................R......................T....S... : 240
*D. rerio* : .D................R......................T....S... : 240
*A. gambiae* : ..................D............................... : 209
*A. mellifera*: ...G...........H..K............................... : 249

 260 * 280 * 300
*A. aegypti* : WSAGCVLAELLLGQPIFPGDSGVDQLVEIIKVLGTPTREQIKEMNPNYTE : 288
*M. musculus* : .................................................. : 290
*H. sapiens* : .................................................. : 290
*D. rerio* : .................................................. : 290
*A. gambiae* : .................................................. : 259
*A. mellifera*: ......................................D........... : 299

* 320 * 340 *
*A. aegypti* : FKFPQIKSHPWQK-------------VFRARTPPDAIALVSRLLEYTPGS : 325
*M. musculus* : .......A...T.-------------...P....E....C........TA : 327
*H. sapiens* : .......A...T.DSSGTGHFTSGVR...P....E....C........TA : 340
*D. rerio* : .......A...T.-------------...P....E....C........TA : 327
*A. gambiae* : .............-------------...T....E............... : 296
*A. mellifera*: .......A.....-------------........E..E..AG......SG : 336

 360 * 380 * 400
*A. aegypti* : RITPIQACAHPFFNELREGNKTLPNGREFPPLFNFTEQELAIQPNLNLIL : 375
*M. musculus* : ..........S......DP.VK.....DT.A.....T...SSN.P.AT.. : 377
*H. sapiens* : ..........S......DP.VK.....DT.A.....T...SSN.P.AT.. : 390
*D. rerio* : ..........S.......P.VK......K.S.....T...SSN.T.AS.. : 377
*A. gambiae* : ...................S.Y......................S..... : 346
*A. mellifera*: ..................QGTR......L........Y..R...S..S.. : 386

 * 420 * 440 *
*A. aegypti* : RPRNP-NDAKAGQSSSSTDGGNSGSGGNGAGANSSTVAGSNSSNNSGTGN : 424
*M. musculus* : I.PHARIQ.A.SPPANA.AAS..NA.DR.QTN.AA.ASA....------- : 420
*H. sapiens* : I.PHARIQ.A.STP.NA.AAS.AN..DR.QTN.AA.ASA....------- : 433
*D. rerio* : I.AHAR.Q.G.STP.NP.ATS.AN..DRSQTTTAA.ASA....T------ : 421
*A. gambiae*  : ....Q-.E.ASKAGQ...-------------------------------- : 363
*A. mellifera*: ...YMQTSEN..GQ.EPVA.SSGNVSD.NVN.TL..SKN..PGQS.MA-- : 434

 460 * 480 * 500
*A. aegypti* : GGSSNSGVAPDSTQGQGGGASGSSQGVGAGAQSVGGAEDITSSQSVPGVD : 474
*M. musculus* : -------------------------------------------------- : -
*H. sapiens* : -------------------------------------------------- : -
*D. rerio* : -------------------------------------------------- : -
*A. gambiae*  : -------------------------------------------------- : -
*A. mellifera*: -------------------------------------------------- : -

 *
*A. aegypti* : SSSSQGAIASAATSTMG : 491
*M. musculus* : ----------------- : -
*H. sapiens* : ----------------- : -
*D. rerio* : ----------------- : -
*A. gambiae*  : ----------------- : -
*A. mellifera*: ----------------- : -

**GSK3 protein sequence is evolutionary conserved between *A. aegypti* and selected organisms.** The percentage of identical residues between *A. aegypti* and the other respective organisms is indicated on the left. The Protein kinases ATP-binding region signature is shown in bold letters with bold underline. The Serine/Threonine protein kinases active-site signature is shown in italic letters with doubly underlined. For clarity, only the N-terminal regions of the proteins were included in the figure but the full-length sequences were included in the alignment.
