# Supplementary material for: Germ band retraction as a landmark in glucose metabolism during Aedes aegypti embryogenesis
Source: BMC Dev Biol. 2010 Feb 25;10:25. doi: 10.1186/1471-213X-10-25 (PMC2838828; doi:10.1186/1471-213X-10-25)
Supplement: Additional file 2 — A. aegypti GSK-3 complete cDNA sequence and primer annealing positions. Representation of the forward and reverse degenerated primers used to clone AeGSK-3 and the primers used for qPCR. [file 1471-213X-10-25-S2.DOC]

25 50 75
ATGAGTGGTCGGCCCAGGACTACCTCTTTTGCGGAGGGCAACAAGACGCAAAACTACCCCGTTATGGGTGGCATG

 100 125 150
AAAATAATCAGTAAAGATGGTTCAAAAGTGACAACGGTTGTGGCAACAGCCGGCCAGGGACCAGACAGACCACAG

 175 200 225
GAAGTATCATACACAGATACCAAGGTGATCGGCAATGGAAGCTTCGGCGTTGTCTTCCAGGCGACGCTCTGTGAT

 250 275 300
ACGGGCGAGCTAGTCGCAATTAAGAAGGTGTTACAGGACAAAAGATTTAAGAACCGCGAACTACAGATTATGCGA

 325 350 375
CGGCTAGAACACTGTAATATTGTTAAACTAAAATATTTCTTTTACTCCAGCGGCGATAAGAAAGACGAAGTTTAT

 400 425 450
CTCAACCTAGTGCTCGAATATATTCCAGAAACCGTATACAAAGTGGCACGTTATTATGCTAAAAACAAACAAACG

 475 500 525
ATACCAATCAATTTTATCAGGCTCTATATGTACCAGCTGTTCCGGAGTCTCGCCTACATCCACTCGCTCGGTATC

 550 575 600
TGCCATCGTGACATCAAACCCCAGAACCTGCTGCTAGACCCGGAGACGGCCGTACTGAAGCTGTGCGACTTTGGC

 625 650 675
AGTGCCAAACAGTTGCTGCATGGGGAGCCGAACGTGTCGTACATCTGCTCGCGATACTACCGGGCACCGGAGTTG

 700 725 750
ATATTTGGTGCCATAAACTATACCACAAAGATCGACGTCTGGAGTGCGGGATGCGTACTAGCCGAATTACTCCTT

 775 800 825
GGACAGCCCATCTTCCCCGGCGACTCCGGCGTCGACCAGTTGGTAGAAATCATCAAGGTCCTCGGCACGCCAACC

 850 875 900
CGGGAACAGATCAAGGAAATGAACCCCAACTATACGGAATTCAAATTCCCCCAGATCAAAAGTCATCCATGGCAG


 925 950 975
AAGGTATTCCGAGCGCGTACCCCACCAGATGCGATCGCACTGGTGTCCCGGTTGCTAGAGTACACGCCGGGATCC

 1000 1025 1050
AGAATAACGCCGATCCAAGCATGTGCGCATCCATTCTTCAACGAGCTACGAGAGGGCAACAAGACACTACCGAAC

 1075 1100 1125
GGACGCGAGTTTCCGCCACTGTTCAACTTCACAGAGCAAGAGCTAGCGATTCAACCAAACCTAAATCTGATACTG

 1150 1175 1200
AGGCCCCGGAACCCGAACGACGCGAAAGCCGGCCAATCGTCCAGCTCGACGGACGGTGGCAACAGTGGCAGCGGC

 1225 1250 1275
GGAAACGGTGCCGGAGCCAACAGCAGCACAGTGGCCGGTAGCAACAGCAGCAACAATAGCGGCACAGGTAACGGT

 1300 1325 1350
GGCAGTAGCAATAGTGGCGTCGCGCCCGACAGCACCCAGGGGCAGGGGGGCGGCGCCAGCGGTAGCAGCCAGGGG

 1375 1400 1425
GTGGGAGCTGGGGCGCAGTCCGTCGGCGGTGCGGAGGATATTACGTCATCACAGTCCGTACCCGGCGTCGACAGT

 1450 1475
AGTTCCAGTCAAGGAGCTATAGCGTCAGCGGCAACTTCTACGATGGGTTAG

***A. aegypti* GSK-3 complete cDNA sequence and primer annealing positions.** The forward and reverse degenerated primers used to clone *Ae*GSK-3 (closed arrows) and the primers used for qPCR (dashed arrows) were, respectively: 5’-GTIGCIATHAARAARGTIYTICARGAY -3’ and 5’- YTTRWRYTCIRTRTARTTIGGRTTCAT -3’; 5’- CGTACATCTGCTCGCGATAC - 3’ and 5’- GGATGCGTACTAGCCGAATT - 3’.
